# A Nomogram for Predicting Survival in Patients With Colorectal Cancer Incorporating Cardiovascular Comorbidities

**DOI:** 10.3389/fcvm.2022.875560

**Published:** 2022-05-27

**Authors:** Hao Wang, Dong Liu, Hanyang Liang, Zhengqing Ba, Yue Ma, Haobo Xu, Juan Wang, Tianjie Wang, Tao Tian, Jingang Yang, Xiaojin Gao, Shubin Qiao, Yanling Qu, Zhuoxuan Yang, Wei Guo, Min Zhao, Huiping Ao, Xiaodong Zheng, Jiansong Yuan, Weixian Yang

**Affiliations:** ^1^Chinese Academy of Medical Sciences and Peking Union Medical College, Fuwai Hospital, National Center for Cardiovascular Diseases, Beijing, China; ^2^Department of Cardiology, Yuncheng Central Hospital, Shanxi Medical University, Yuncheng, China; ^3^Department of Oncology, Yuncheng Central Hospital, Shanxi Medical University, Yuncheng, China; ^4^Department of Oncology, Yunnan Cancer Hospital, Kunming, China; ^5^Department of Oncology, Jiangxi Cancer Hospital, Nanchang, China; ^6^Department of Oncology, Chongqing Cancer Hospital, Chongqing, China; ^7^Key Laboratory of Pulmonary Vascular Medicine, Fuwai Hospital, National Center for Cardiovascular Diseases, Chinese Academy of Medical Sciences and Peking Union Medical College, Beijing, China

**Keywords:** colorectal cancer, comorbidity, cardiovascular disease, prognosis, nomogram

## Abstract

**Background:**

Cardiovascular comorbidities (CVCs) affect the overall survival (OS) of patients with colorectal cancer (CRC). However, a prognostic evaluation system for these patients is currently lacking.

**Objectives:**

This study aimed to develop and validate a nomogram, which takes CVCs into account, for predicting the survival of patients with CRC.

**Methods:**

In total, 21,432 patients with CRC were recruited from four centers in China between January 2011 and December 2017. The nomogram was constructed, based on Cox regression, using a training cohort (19,102 patients), and validated using a validation cohort (2,330 patients). The discrimination and calibration of the model were assessed by the concordance index and calibration curve. The clinical utility of the model was measured by decision curve analysis (DCA). Based on the nomogram, we divided patients into three groups: low, middle, and high risk.

**Results:**

Independent risk factors selected into our nomogram for OS included age, metastasis, malignant ascites, heart failure, and venous thromboembolism, whereas dyslipidemia was found to be a protective factor. The c-index of our nomogram was 0.714 (95% *CI*: 0.708–0.720) in the training cohort and 0.742 (95% *CI*: 0.725–0.759) in the validation cohort. The calibration curve and DCA showed the reliability of the model. The cutoff values of the three groups were 68.19 and 145.44, which were also significant in the validation cohort (*p* < 0.001).

**Conclusion:**

Taking CVCs into account, an easy-to-use nomogram was provided to estimate OS for patients with CRC, improving the prognostic evaluation ability.

## Introduction

Colorectal cancer (CRC) is the third most common cancer and the fifth most common cause of cancer-related deaths (8.6%) in China, which imposes a heavy burden on the healthcare system (1, 2). Increasing evidence suggests that CRC and cardiovascular comorbidities (CVCs) are not separate disease entities, and studies have described the shared pathophysiology between CRC and CVCs (3). Patients with CRC are at 2–4 times increased risk of developing cardiovascular diseases (4), and CVCs are the leading cause of death among patients with CRC (5), which due to the fact that CVCs not only increase the non-cancer mortality but also restrict the options for treatment. Therefore, the OS of patients with CRC is not only influenced by cancer-related factors but also closely related to CVCs. However, the prognostic models combined with CVCs factors for patients with CRC are still lacking.

The TNM staging system of the 8th edition of the American Joint Committee on Cancer (AJCC) is widely used in prognosis prediction for patients with CRC. However, it may be inapplicable to patients with CVCs because it only includes cancer-related variables (6). To integrate CVCs and cancer-related prognostic factors for providing more individualized risk estimates, a nomogram, which could estimate numerical probabilities for individual patients by incorporating prognostic factors (7, 8), can be used to develop a more individual and more accurate prognostic tool.

In this study, we aimed to develop and validate a prediction model, which was visualized as a nomogram, for the OS of patients with CRC and CVCs, and stratify patients into three risk groups. This nomogram may provide a more individualized prognosis for these patients and guide the selection of treatment regimens.

## Patients and Methods

### Patients Selection and Predictor Variables

Data present in this study were collected through a computer-assisted personal interview system from January 2011 to December 2017. We collected information on newly diagnosed primary CRC inpatients from four cancer specialized hospitals in China (Yunnan Cancer Hospital, Jiangxi Cancer Hospital, Chongqing Cancer Hospital, and Yuncheng Central Hospital). The data for birthdays, gender, diagnosis dates, and the information of diagnosis were abstracted from hospital discharge records. Each record had information on up to 30 diagnoses, which were used to identify comorbidities of patients. The follow-up information was collected by clinic visit, hospitalization, or telephone call.

The exclusion criteria were as follows: (1) patients who were under the age of 18 or over the age of 90; (2) pathologically confirmed benign cancers or cancer-like diseases; (3) patients with more than one cancer; and (4) patients who had incomplete data. A total of 21,432 patients were selected in the final dataset. We divided the dataset into a training cohort (19,102 patients, from Yunnan, Jiangxi, and Yuncheng) and a validation cohort (2,330 patients from Chongqing). The study was conducted in accordance with the Institutional Review Board of the participating hospitals and informed consent was waived because the data were de-identified.

The CVCs in our study defined as the pre-existing cardiovascular diseases when cancer was diagnosed included: hypertension, diabetes, coronary artery disease, heart failure, dyslipidemia, atrial fibrillation, cerebrovascular disease, pericardial effusion, and venous thromboembolism. The cancer-related variables in our study included: age of diagnosis, gender, metastasis (M stage in TNM staging system, M1a denotes metastasis to one distant site or organ, M1b denotes metastasis to more than one, and M1c for peritoneal metastasis), malignant pleural effusion, and malignant ascites. Continuous variables were translated into categorical variables.

### Statistical Analysis

Statistical analysis was performed using SPSS 22.0 (IBM, Chicago, IL, United States) and R version 4.4.0.^1^ Overall survival (OS) was defined as the time from the cancer diagnosis to the patient’s death or censored at the time of the last follow-up. Variables were screened by univariate Cox regression analysis (*p* < 0.10), and every variable of CVCs and cancer was included in the process of variables screening. Variables selected from the screening process were subjected to a multivariable Cox regression analysis model (“nomogram model”) which was used to develop the nomogram. Furthermore, the backward stepwise and forward stepwise methods were used to identify the combination of variables, and the two methods were compared by the value of the Akaike information criterion (AIC). We examined the proportional hazard assumption by plotting the log minus log survival curves and found it to hold. A decision curve analysis (DCA) was conducted to determine the clinical usefulness of the prediction model by quantifying the net benefits at different threshold probabilities (9). To evaluate the significance of adding CVCs into the CRC prognostic scoring system, the multivariable Cox regression model which only included cancer-related variables (“cancer model”) was developed, it was used in DCA to compare with the nomogram model.

The discrimination and calibration of the nomogram model were assessed by the concordance index (c-index) and calibration curve which were subjected to 1,000 bootstrap resamples for internal validation, and the validation cohort was used in external validation. The clinical utility of the “nomogram model” and “cancer model” was compared by DCA. We calculated the nomogram score of each patient and used the x-tile software (a bioinformatics tool for outcome-based cut-off value optimization) (10) to divide patients into three groups: low risk, middle risk, and high risk. The Kaplan–Meier method and log-rank test were used for survival analysis. All statistical tests were two-tailed, and the value of *p* < 0.05 was considered to be statistically significant.

## Results

### Basic Characteristics

A total of 19,102 patients with CRC were included in the training cohort, the mean age was 59.06 ± 12.33 years with 57.2% male, and 7,124 (37.3%) patients died of all causes (Table 1). The validation cohort included 2,330 patients and 815 (35.0%) patients died during the follow-up (Table 1). Patients in the validation cohort were older (61.01 ± 13.06) with a higher proportion of men (58.7%) and a higher prevalence of CVCs.

**TABLE 1:** Baseline clinical features.

Characteristics	Training cohort (*n* = 19,102)	Validation cohort (*n* = 2,330)
Age, year	59.06 ± 12.33	61.01 ± 13.06
**Age stratification, year**		
<50	4,106 (21.5%)	477 (20.5%)
50–59	5,478 (28.7%)	477 (20.5%)
60–69	5,497 (28.8%)	732 (31.4%)
70–79	3,298 (17.3%)	487 (20.9%)
≥80	723 (3.8%)	157 (6.7%)
**Gender**		
Male	10,925 (57.2%)	1,368 (58.7%)
Female	8,177 (42.8%)	962 (41.3%)
**T stage*^a^***	*n* = 3,180	*n* = 545
T1	80 (2.5%)	27 (5.0%)
T2	359 (11.3%)	118 (21.7%)
T3	1,090 (34.3%)	204 (37.4%)
T4	1,650 (51.9%)	196 (36.0%)
**N stage*^a^***	*n* = 3,180	*n* = 545
N0	1,608 (50.6%)	258 (47.3%)
N1	773 (24.3%)	174 (31.9%)
N2	799 (25.1%)	113 (20.7%)
**Metastasis**		
M0	12,984 (68.0%)	1,357 (58.2%)
M1a	4,225 (22.1%)	743 (31.9%)
M1b	1,654 (8.7%)	203 (8.7%)
M1c	239 (1.3%)	27 (1.2%)
Pleural effusion	222 (1.2%)	44 (1.9%)
Malignant ascites	446 (2.3%)	93 (4.0%)
CVCs	4,148 (21.7%)	739 (31.7%)
Hypertension	2,614 (13.7%)	383 (16.4%)
Diabetes	1,282 (6.7%)	219 (9.4%)
Coronary artery disease	388 (2.0%)	124 (5.3%)
Dyslipidemia	34 (0.2%)	92 (3.9%)
Heart failure	108 (0.6%)	114 (4.9%)
Atrial fibrillation	69 (0.4%)	20 (0.9%)
Cerebrovascular disease	789 (4.1%)	118 (5.1%)
Venous thromboembolism	120 (0.6%)	48 (2.1%)
Pericardial effusion	46 (0.2%)	4 (0.2%)

*Data are presented as mean ± SD or No (%).*

*CVCs, cardiovascular comorbidities.*

*^a^3,725 patients had records of T stage and N stage.*

### Variables Selection and Prediction Model Development

**FIGURE 1:**
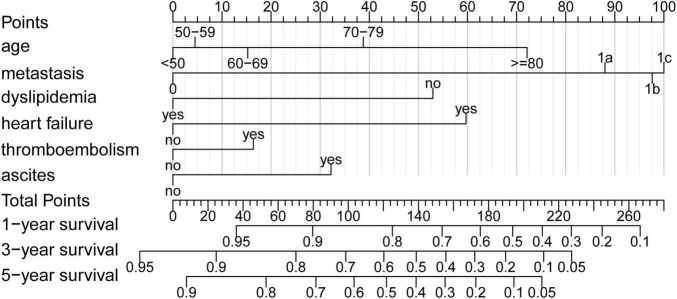
Nomogram. Nomogram to predict the probability of 1-, 3-, and 5-year overall survival (OS) in patients with colorectal cancer (CRC). In addition, 1-, 3-, and 5-year OS could be obtained by adding up the points of each corresponding variable.

All variables except gender were selected using the univariable Cox regression analysis (Table 2) and were subjected to the multivariable Cox regression analysis. The backward stepwise method performed better than forward stepwise method in AIC (AIC for backward stepwise was 131,341.6, which was smaller than 131,355.2 for the forward stepwise). The result showed that age, metastasis, malignant ascites, dyslipidemia, heart failure, and venous thromboembolism had important predictive value for the prognosis of patients with CRC (Table 2). Interestingly, dyslipidemia, as a traditional risk factor for cardiovascular disease, was found to be a protective factor. In addition, patients with at least one kind of CVC have a higher risk of death than patients without (hazard ratio [*HR*] = 1.194 (1.130–1.261), *p* < 0.001, Table 2). The nomogram (Figure 1) was developed based on multivariable Cox regression analysis. By calculating the total point of a patient, and drawing a vertical line from the total point axis to three outcome axes, estimated 1-, 3-, and 5-year survival probabilities could be obtained.

**TABLE 2:** Univariate and multivariate cox regression analysis between characteristics and overall survival (OS).

Characteristics	Univariate analysis		Multivariate analysis	
	HR (95%CI)	*p*-value	HR (95%CI)	*p*-value
**Age stratification, year**		<0.001		<0.001
<50	Reference		Reference	
50–59	1.002 (0.934–1.075)	0.957	1.076 (1.003–1.154)	0.041
60–69	1.129 (1.054–1.209)	0.001	1.283 (1.197–1.375)	<0.001
70–79	1.489 (1.384–1.602)	<0.001	1.887 (1.753–2.032)	<0.001
≥ 80	2.300 (2.065–2.562)	<0.001	3.252 (2.916–3.627)	<0.001
**Gender**				
Male	Reference			
Female	1.006 (0.959–1.054)	0.818		
**Metastasis**		<0.001		<0.001
M0	Reference		Reference	
M1a	4.068 (3.861–4.286)	<0.001	4.234 (4.015–4.464)	<0.001
M1b	4.789 (4.477–5.122)	<0.001	4.956 (4.626–5.309)	<0.001
M1c	5.612 (4.833–6.517)	<0.001	5.147 (4.397–6.025)	<0.001
Pleural effusion	3.187 (2.740–3.706)	<0.001		
Malignant ascites	3.664 (3.291–4.080)	<0.001	1.686 (1.505–1.890)	<0.001
Hypertension	1.096 (1.026–1.172)	0.006		
Diabetes	1.085 (0.991–1.188)	0.078		
Coronary artery disease	1.248 (1.071–1.455)	0.005		
Dyslipidemia	0.414 (0.186–0.921)	0.031	0.418 (0.188–0.932)	0.033
Heart failure	5.513 (4.532–6.705)	<0.001	2.572 (2.111–3.135)	<0.001
Atrial fibrillation	1.372 (0.970–1.942)	0.074		
Cerebrovascular disease	1.303 (1.168–1.454)	<0.001		
Venous thromboembolism	2.072 (1.640–2.617)	<0.001	1.308 (1.034–1.653)	0.025
Pericardial effusion	3.000 (2.152–4.182)	<0.001		
CVCs*^a^*	1.194 (1.130–1.261)	<0.001	–	

*^a^Patients have at least one kind of cardiovascular comorbidities.*

### Validation of the Prediction Model

**FIGURE 2:**
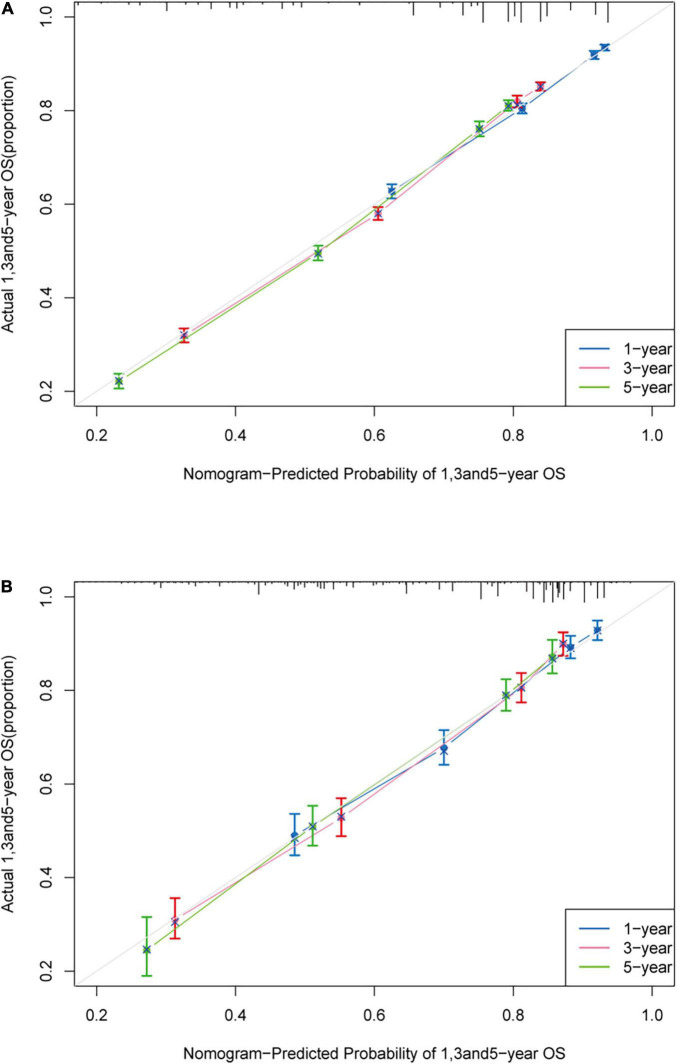
Calibration plot. Calibration curve of the nomogram both in the training and validation cohort. Predicted survival probability produced by nomogram is *x*-axis, and actual survival is *y*-axis, close alignment with 45 degrees diagonal represents the good estimation. **(A)** 1-, 3-, and 5-year OS of the training cohort; **(B)** 1-, 3-, and 5-year OS of the validation cohort.

The c-index of the training cohort was 0.714 (95% *CI*: 0.708–0.720) while in the validation cohort was 0.742 (95% *CI*: 0.725–0.759), which indicated acceptable discrimination. The calibration curves in the training cohort and validation cohort were closely aligned with the 45 degrees diagonal. It revealed good concordance between the nomogram predicted probabilities and the observed probabilities (Figure 2).

### Decision Curve Analysis

**FIGURE 3:**
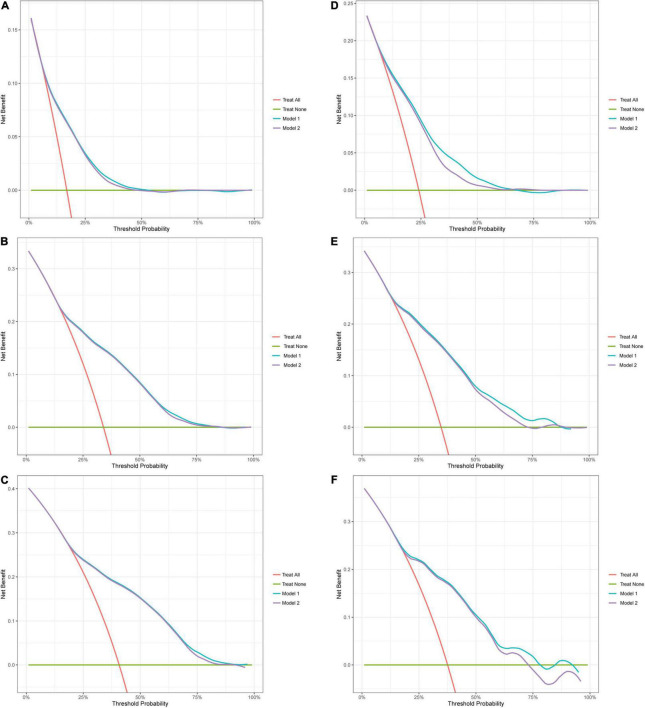
Decision curve analysis (DCA). Decision curve analysis for OS. Red line (Treat all): “all patient dead scheme.” Green line (Treat none): “no patient dead scheme.” Blue line (Model 1): “nomogram model.” Purple line (Model 2): “cancer model.” **(A)** 1-year DCA in the training cohort; **(B)** 3-year DCA in the training cohort; **(C)** 5-year DCA in the training cohort; **(D)** 1-year DCA in the validation cohort; **(E)** 3-year DCA in the validation cohort; **(F)** 5-year DCA in the validation cohort.

In DCA (Figure 3), the net benefit of the decision curves for the “nomogram model” is higher than all patient dead scheme or no patient dead scheme. Furthermore, the “nomogram model” was constantly higher in net benefit compared with the “cancer model.” The net benefit was comparable. It demonstrated that our nomogram in predicting OS is more beneficial than that of only including cancer-related variables.

### Risk Stratification of Overall Survival by the Prediction Model

**FIGURE 4:**
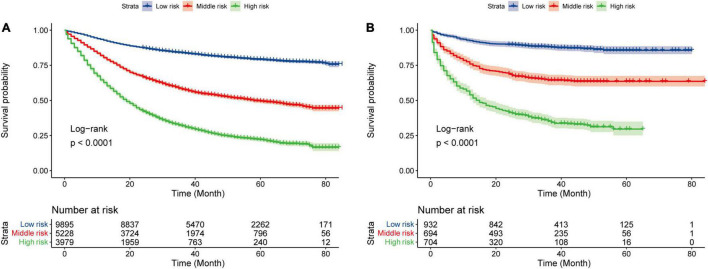
Kaplan–Meier curves for three groups. Kaplan–Meier curves for three groups in the training cohort **(A)** and the validation cohort **(B)**.

We divided patients into three groups: low risk (0–68.19), middle risk (68.19–145.44), and high risk (>145.44) according to their nomogram scores by x-tile software (10). The survival curve of the training cohort and validation cohort is shown in Figure 4. To verify the reliability of the cutoff value, log-rank tests were used to compare survival between three groups (*p* < 0.001, the Bonferroni-corrected level of significance in this analysis was *p* < 0.0167).

## Discussion

The information of 21,432 patients with CRC cancers was collected in our study. We developed and validated a nomogram, which combined cancer and CVC variables, for survival prediction. Compared with only focusing on cancer-related variables, the addition of CVCs variables could provide individual prognostic information and guide clinical decision-making. To our knowledge, this is the first nomogram assessing the prognostic impact of CVCs in patients with CRC.

With the differences in comorbidities definitions, study populations, and cancer types, it is difficult to state with certainty how common the CVCs are (5). However, our result showed that the prevalence of CVCs in patients with CRC was higher than in the general population (Table 1) (11). Although the effects of different CVCs on patients with CRC are different, CVCs make the risk of death in patients with CRC rise by 19.4% relatively (Table 2). We found heart failure and venous thromboembolism were the risk factors for the prognosis of patients with CRC, whereas dyslipidemia was a protective factor.

In recent years, various prognostic models of CRC have been described (12–15). Despite these models being used in different clinical scenes, age and metastasis (or M stage) can be found in almost all models. It is known that the OS of patients with CRC declines with age (16), and that is consistent with our study. The effect of age on the prognosis is increasing with age, which can be seen in the increasing score interval of age stratification in our nomogram. The peritoneal metastasis of patients with CRC, which has a poor prognosis, is often viewed as a preterminal state reflecting widespread cancer dissemination (17). For this reason, the M stage has been expanded in the 8th edition of the AJCC TNM staging system (adding M1c for peritoneal metastasis) (8). However, an analysis showed that in 72 clinical trials of metastatic CRC, only seven trials include peritoneal metastasis (18). Peritoneal metastasis as a prognostic indicator has rarely been included in previous studies. We extracted the metastatic sites from the diagnostic information in our database and demonstrated that the prognosis of patients becomes poor with the rise of the M stage. It should be noted that the gold standard to assess peritoneal metastasis is operative exploration, and for patients who did not undergo surgery, conventional imaging examinations, such as computerized tomography (CT) lack the resolution to detect early peritoneal metastasis (19), thus the incidence of peritoneal metastasis may be underestimated. Furthermore, the most common cause of malignant ascites is peritoneal dissemination, which accounts for approximately 53% of cases (20), although the presence of malignant ascites is an apparent poor prognostic factor (21), previous prognostic prediction models rarely incorporate this variable. Our study showed that malignant ascites as a predictor of prognosis cannot be neglected.

Heart failure, which has the highest score among CVCs in our nomogram, is one of the most significant risk factors for prognosis in patients with CRC. It is consistent with the study of Gross et al., which showed that about 9% of patients with stage I–III colorectal cancer died due to heart failure, and heart failure was the most significant comorbidity affecting the OS (22). Heart failure can not only increase the non-cancer mortality for patients with CRC (23) but also restrict the options for treatment and reduce patients’ compliance (24, 25). Despite advances in management, heart failure still has a worse prognosis than some of the common cancers in both men and women (26). Furthermore, our study shows that 85.2% of heart failure patients entered the high-risk group, and the OS of patients with heart failure is significantly shorter than that of patients without heart failure [median: 10.0 (Q1: 1.0, Q3: 30.2) vs. median: 36.0 (Q1: 21.0, Q3: 52.0), *p* < 0.001].

Cancer is a significant risk factor for venous thromboembolism (VTE). Thrombotic events sometimes may become the first manifestation of cancer (27). Research shows that the 2-year cumulative incidence rate of VTE after cancer diagnosis was 3.1% (28). However, autopsy studies have confirmed the occurrence of pulmonary embolism in patients with CRC was as high as 28% (29). This means that many of the thrombotic events in patients with CRC are not detected because of the accuracy of examination and the unremarkable clinical manifestations. The relatively low incidence of thrombotic events in our cohort is also related to this reason. VTE is the second leading cause of death in patients with cancer after cancer progression (30). The development of VTE in patients with cancer reflects their enhanced cancer-associated thrombin generation, which demonstrates that the cancer is biologically more aggressive (31). Therefore, for patients with VTE or with high thrombotic risk based on predictive models [such as, the Khorana risk scoring model (32) and the COMPASS-CAT risk assessment model (33)], rational intervention for the VTE could improve these patients’ outcomes (34).

However, the interaction between CVCs and cancer is sometimes protective. Although some studies on the relationship between dyslipidemia and cancer considered dyslipidemia as a protective factor (35, 36), the results of different CRC studies conducted to date have been inconsistent (37, 38). Our study identified that dyslipidemia was a protective factor. Research showed that serum lipids, especially cholesterol, are involved in many processes of cancer development (39) and make up the lipid rafts in the cancer cell membrane, which are involved in the transduction of signaling pathways related to the cancer cell survival (40). To meet the increasing need for cholesterol, the process of cholesterol absorption is enhanced in cancer cells, which may lead to a decreased cholesterol concentration in patients with CRC (41). Furthermore, the changed concentration of serum lipid might be related to the malignancy of CRC. Zhang et al. have found that with the progression of the TNM stage in patients with CRC, the concentration of serum total cholesterol and triglyceride were reduced significantly (42). These results showed the negative correlation between the concentration of serum lipid and the severity of CRC, and dyslipidemia could thus reflect the prognosis of patients with CRC indirectly. However, more studies are still needed to validate the impact of various lipid biomarkers on the prognosis of CRC.

The TNM staging system is still the “golden standard” for CRC prognosis in clinics. However, as a classifier that groups patients into ordered risk strata, the lack of CVCs factors caused its inability to deal with heterogeneity within risk groups caused by CVCs. Thus, the risk calculators (such as nomograms), which could utilize multiple prognostic factors to provide more individualized risk estimates, gained increasing popularity. To improve the quality and acceptability of the risk models for patients with cancer, AJCC has put forward the acceptance criteria for risk models for individualized prognosis (43). However, of the 29 published risk calculators for colon or rectal cancer, only 3 have been endorsed by the AJCC (8), and the models focused on CVCs are still lacking. Therefore, in this study, we followed these acceptance criteria and developed an easy-to-use nomogram, which included the variables of cancer and CVCs that are readily available in clinical work and most relevant to the prognosis for patients with CRC and CVCs. In the following work, we will work to compare the availability of our nomogram and TNM staging system in guiding the choice of patients’ treatment options.

### Limitation

Although our study had a large sample size, there were still some limitations. First, we could not be sure of the causal relationship between CVCs and CRC because the time of CVCs emerging was not recorded, so the impact of CVCs on the prognosis of patients with CRC was our point. Second, the therapeutic regimens of CRC developed rapidly during the data collection period, but the database lacked detailed treatment regimens. Considering that some anticancer therapies potentially have cardiotoxicity, adding more treatment-related variables to the nomogram or developing different nomograms based on different therapies is meaningful. Third, our nomogram did not include molecular or genetic biomarkers that had prognostic value. However, considering the high cost of biomarkers tests, our nomogram may also be easier to apply in the clinic.

## Conclusion

In this study, two CVCs and three cancer-associated characteristics were identified as risk factors for the prognosis of patients with CRC, whereas dyslipidemia exerted a protective effect. Taking CVCs into account, we developed and validated a nomogram that could estimate OS for patients with CRC. It may improve the prognostic evaluation ability and facilitate individualized patient management.

## Data Availability Statement

The original contributions presented in the study are included in the article/supplementary material, further inquiries can be directed to the corresponding authors.

## Ethics Statement

This study was reviewed and approved by and conducted in accordance with the Institutional Review Boards of Yunnan Cancer Hospital, Jiangxi Cancer Hospital, Chongqing Cancer Hospital, and Yuncheng Central Hospital. The requirement of written informed consent was waived because the data were deidentified.

## Author Contributions

HW and DL: conception of design and analysis, and interpretation of data. HW, DL, JSY, WY, YQ, ZY, WG, MZ, HA, XZ, SQ, XG, JGY, HX, JW, TW, TT, HL, ZB, and YM: data collection and drafting of the manuscript or revising it critically for important intellectual content. JSY and WY: final approval of the manuscript submitted. All authors contributed to the article and approved the submitted version.

## Conflict of Interest

The authors declare that the research was conducted in the absence of any commercial or financial relationships that could be construed as a potential conflict of interest.

## Publisher’s Note

All claims expressed in this article are solely those of the authors and do not necessarily represent those of their affiliated organizations, or those of the publisher, the editors and the reviewers. Any product that may be evaluated in this article, or claim that may be made by its manufacturer, is not guaranteed or endorsed by the publisher.

## Footnotes

1
http://www.r-project.org/

## Abbreviations

CVCs: cardiovascular comorbidities
CRC: colorectal cancer
OS: overall survival
C-index: Harrell’s concordance index
DCA: decision curve analysis
CI: confidence interval
HR: hazard ratio.
